# Marriage—Should a Certificate of Health Be Required before Issuing a License to Marry?

**Published:** 1912-10

**Authors:** Theo. Y. Hull

**Affiliations:** San Antonio, Texas, Secretary State Society of Social Hygiene


### Marriage—Should a Certificate of Health Be Required Before Issuing a License to Marry?

BY THEO. Y. HULL, B. S., M. D., SAN ANTONIO, TEXAS,
Secretary State Society of Social Hygiene.

"We can not recreate what is dead, we can not stop the march of events, but we can direct this march and out of the new conditions develop something better than the past knew."—*Roosevelt.*

Speer, in his treatise on the "Law of Married Women in Texas," says marriage "is an institution established by God Himself, is recognized by all Christians and civilized nations, and is essential to the peace, happiness and well-being of society." It is not treated as a mere contract, but as a relation or a condition, resulting from a civil contract between one man and one woman of the needful civil and physical capacity. The underlying idea from the legal viewpoint, however, is the ability of the parties to fulfill the agreement, and consequently the legal enactments governing the marriage contract are not essentially different from those concerning civil contracts in general. The rule is that every person who is capable of contracting, and who is not forbidden by statute, may enter into the marriage relation. Certain persons, however, are forbidden to marry by statutory enactment.

The power to control or regulate the marriage of its domiciled citizens is one which every sovereign nation in the civilized world exerts. In the United States, each State has full power to pass laws governing marriage. In the State of Texas, as in practically every State, certain restrictions are placed upon it. In the main, these are the restrictions that have been enforced in most countries for many decades.

The law requires that the parties contracting to marry must have sufficient mental capacity to understand the marriage relation. Thus an idiot or a lunatic would not be considered competent to contract to marry, for the same reason that he or she is not competent to make any other valid agreement, or understand the same. In other words, the law demands that the parties at the time of contract shall have the capacity to give intelligent consent. A person may have insane delusions or impulses, and yet in the eyes of the law be competent to marry. The law also stipulates that males under 16 and females under 14 may not marry, and it declines to ratify the marriages, either of both of whom are under the age of consent. The law deems such parties incapable of intelligent consent. In both of these restrictions the law seeks to protect property rights which may be important and far-reaching. The basic idea is one of public policy in regard to property which either or both may possess.

The law likewise prohibits the intermarriage of whites with blacks, and *vice versa*. Such marriages are repugnant to intelligent people and against good public policy. Consanguinity and affinity are canonical disabilities. The law prohibits the marriage of such parties if the relationship be within the prescribed degrees. This restriction is in deference to the church and public expediency. A fifth restriction prohibits polygamy.

But marriage is more than an agreement between competent parties. It is an agreement between one man and one woman to live together as man and wife with the implied object of begetting offspring. It is the legalized method of propagating the race. It is a custom older than the law, but a custom which society in its evolution has sought to protect by adding such safeguards as it has deemed wise.

The legal restrictions relating to mental capacity, age, race and consanguinity were not imposed because of any thought of what might or might not be the effect upon the offspring resulting from the union. The idea that the offspring of those too immature in years might not be healthful and vigorous, and that those mentally defective might beget children that would inherit the parental defect, become idiots or imbeciles, mental perverts, criminals or insane, did not enter into the law's conception of marriage. The underlying idea was not defective offspring. Only one restriction may be construed as due to any thought of the child. Custom, through a long period of evolution, has written into our statute books the restriction that binds together one man and one woman in the bonds of matrimony. In the early stages of society the battle was to the strong, both physically and numerically. It was found that polygamy favored disease and sterility. It was found that in such tribes the offspring decreased in numbers, and were physically weaker. On the other hand, it was found that among those tribes practicing monogamy the offspring were both more numerous and physically more vigorous. The stronger tribes conquered the weaker, and imposed upon them their customs.

Up to the last few decades the conditions of human life were such that there appeared little need to consider measures looking to its conservation. Neither was the public mind awake to the need of such action. But events have come rapidly in the last few years. The conditions under which we live have changed.

More than one-half of the population has concentrated in the cities. We are face to face with some very grave problems concerning the perpetuity and efficiency of the race itself. The great conservation movement which was ushered in a few years ago is now attempting the solution of these problems. The people have to a degree been aroused to the necessity of protecting themselves and their offspring from the encroachment of disease. They are beginning to realize that crime, degeneracy and insanity are on the increase. They have learned that heredity plays a very important part in human affairs. All over the country these forces are uniting and concentrating their attention upon certain questions. One of these is the protection of the home.

Viewing the marriage relation from the standpoint of the offspring, the wife, the home and public health, is there no necessity for further safeguarding that most sacred of all human relations? In the past we have learned certain things by hard experience. Shall we learn yet other lessons in the same way, or will we apply to human race culture the knowledge we now possess? This is the practical question of today.

Shall we consider everyone competent to marry who meets the mental requirements, who is of sufficient age, who fulfills the race condition, who is not too near of kin and who is willing to be content with monogamy? Shall the epileptic, although he fulfills every one of the requirements, be permitted to marry and beget children that may be epileptic, degenerates, moral perverts, or insane? Shall the chronic inebriate and the drug habitue marry and bring into existence offspring of such lowered nervous equilibrium that many become nervous bankrupts, some epileptic, and others insane? Shall the syphilitic marry, destroy the health and happiness, if not the life, of the one he has sworn to protect, and bring into this world deformed, defective and deficient offspring? Shall the victim of gonorrhea marry, put out the eyes of his child, and condemn his wife to future barrenness, destroy her health, and bring her ultimately to the operating table? Shall the one with open tuberculosis marry, infect the healthy and beget children whose lives will be sacrified on the same altar as the parents? God forbid, and public policy forbid. If marriage be an institution ordained of God, then man has been appointed to guard its portals and keep the sanctuary inviolate and pure.

There are those who will not believe that the existing conditions warrant any attempt to further regulate marriage or guard the marriage relation. There are those who profess to believe that the wife must take her chances for better or for worse. There are those who seem to consider that the unborn child has no implied rights to a good inheritance. There are those who think that the State has no vital interest in the physical and mental efficiency of its citizenship. There are those also who profess to see in the application of the principles of eugenics to the end that the epileptic, the chronic inebriate and drug habitue, and those suffering with such disease as uncured syphilis, open tuberculosis, and gonorrhea, something "unphilosophical, unscientific and impracticable."

If the disease ended with the infected one and entailed no further evils than the resulting misery and suffering of the individual and cost to the State for the many thus rendered incapable of self-support, there would still be ample reason to prohibit such a one from entering into the marriage relation, but the train of evils does not end with the diseased one, the innocent party to the contract suffers in a vast majority of the cases, the offspring almost invariably suffer lowered vitality, deformity, mental deficiency, or other signs of an abnormal nervous system. From such marriages, largely, springs that large and ever-increasing class of paupers, delinquents, moral perverts, and criminals of various degrees.

Ten persons in every three thousand are insane; six of these cases are of the inherited type. In other words, 60 per cent give a family history of insanity, epilepsy, or kindred disorders. The children of epileptics may be epileptic or insane. The advice of Dr. Holmes to the parents of a defective child that a consultation should have been held fifty years before the child was born is pertinent here. Congenital defects are largely due to parental defects. Dr. Frederick W. Mott, F. R. S., of England, in his report to the Royal Society of Medicine, says: "The more care that is taken in going into the history of the stock, direct and collateral, the more convincing will be the evidence of the importance of heredity in the production of all disease." The history of a family cited by Dr. Mott can be duplicated by a very large per cent of physicians. In this family, the first generation, the father was normal, but the mother committed suicide. Their only child, a son, married a woman whose mother was insane. The husband committed suicide and the wife became insane. Four children were born to this pair. All became insane later. In the next generation one of the preceding four, a daughter, married a normal man. The wife was epileptic, became insane and committed suicide. One child was the result of this union. This child became insane and committed suicide. The family history ends with the fourth generation, when, fortunately, the last member died.

In the case of tuberculosis, Squires reports that in England 24.6 per cent of children of non-tuberculous parents contract the disease, against 33.15 per cent among children of tuberculous parents. Dr. Sachs, of Chicago, in speaking of his studies of the prevalence of tuberculosis among the children of the laboring classes, says that, of the living children of tuberculous parents, 53.1 per cent are infected. Tuberculosis is so serious a malady that we should use every possible means of combating its spread. The issuing of a license to marry to a party with open tuberculosis is equivalent to consenting to the death of the innocent party, and ignores the rights of the unborn.

The prevalence of the venereal diseases, like tuberculosis, is almost beyond the comprehension of sane people. These are the diseases so frequently spread by marriage, and are consequently so important to this subject. Dr. Prince A. Morrow places the number of syphilitics in the United States at 2,000,000. He says further that the extermination of the social diseases (syphilis and gonorrhea) would mean the elimination of at least one-half of our institutions for defectives, and that the blighting, destructive effect of syphilis upon the offspring is enormous. Competent judges believe the social diseases to be the most powerful factors in the degeneration and depopulation of the world. Neisser, a distinguished German authority, states that 15 per cent of the male population has syphilis. Probably 15 per cent or more of our inexcusably high infant mortality is due to its blighting effects upon the offspring.

According to Neisser, fully 75 per cent of the adult male population contracts gonorrhea. This is a terrible indictment of the manhood of the country. Probably 500,000 young men in this country contract one of the venereal diseases each year. If even one-fourth of these marry, think of the devastation that is wrought. In some instances death mercifully intervenes. In others a life of suffering is the penalty. In still others the divorce courts settle the question, and its harvest is great.

These effects are not imaginary. Even with our imperfect system of vital statistics, we have been able to determine a part of the evil wrought. It is now known that gonorrheal ophthalmia is responsible for from 25 to 40 per cent of all blindness. The State of New York has 6200 totally blind people, 32 per cent whom are sightless from this one disease. Over 33 per cent of the children admitted into the Pennsylvania School for the Blind are blind from this cause. In New York State one child in every 200 born is blind from gonorrheal infection. Nor is this all, there are many more with greater or less destruction of sight, lowering their efficiency just to that degree. It is estimated that every person blind from birth costs the State $10,000. The State thus lays millions on the altar of "personal license," sometimes mistakenly called "personal liberty."

Blindness is but one of the results of gonorrhea. It is now known that much of the sterility in women (50 per cent)—the childless marriages—is due to it, that though some escape with only the capacity to bear children destroyed, many wives thus innocently infected become chronic invalids and ready victims to intercurrent diseases, and more, that fully 50 per cent of all abdominal operations performed on women are due to this cause alone. (Some authorities insist that gonorrhea causes 80 per cent of all inflammatory diseases in women and 60 per cent of gynecological operations.)

Now it is proposed to ask for legislation in this State to protect the innocent party to the marriage contract, in so far as possible from the blighting effect of these diseases. We do not expect all of these evils to be corrected by such legislation, or even a large per cent of it, but we do expect much good to come from compelling each party to the marriage contract to present satisfactory evidence that he or she is not suffering from disease of the character mentioned.

Texas has always been prompt in seeking advanced legislation, but the people of many other States are demanding of their legislative bodies protection in these very matters. Some States have already enacted such measures. Connecticut in 1895 enacted a measure prohibiting the marriage of epileptics, imbeciles and feeble-minded persons. The New York Legislature on March 5, 1912, passed a bill designated as "An act to amend the public health law, in relation to operations for the prevention of procreation" in certain criminals. The Oregon Legislature passed a bill requiring a physical examination before marriage, but it was vetoed by the Governor. A bill was introduced in the Indiana Legislature prohibiting "county clerks from issuing a license to marry to any male who fails to present a medical certificate showing him to be free from all venereal diseases," but it failed to pass. Similar bills were introduced in the Legislatures of North Dakota, Wisconsin, Ohio and Rhode Island. There was considerable agitation in Vermont and Massachusetts. California expects to enact a law at the next session of the Legislature.

Dr. S. G. Dixon, Commissoner of Health for Pennsylvania, says: "I think it one of the most important preventative measures that can be raised on the health laws of any government."

Dr. Oscar Dowling, President of the Louisiana State Board of Health, in his public addresses, has taken "especial pains to refer to the evil effects of gonorrhea, syphilis, tuberculosis and other diseases which are liable to affect the offspring." He says: "I believe the time has come when people should be required to produce health certificates before they be allowed to marry." Many other State Health Officers have expressed themselves in a similar way, and only one has opposed such legislation.

We are now face to face with this proposition. Will the State protect innocent women and unborn children by enacting a law requiring a medical certificate of health before granting a license to marry to any one, male or female? Will the people demand this protection? Do the lines of Kipling apply to our condition:

"Men are not moved to better things
By wit or common sense,
But cuffed by priests and kicked by kings,
Use them in self-defense."

